# Involvement of Chromatin Remodeling Genes and the Rho GTPases *RhoB* and *CDC42* in Ovarian Clear Cell Carcinoma

**DOI:** 10.3389/fonc.2017.00109

**Published:** 2017-05-29

**Authors:** Nicolai Skovbjerg Arildsen, Jenny-Maria Jönsson, Katarina Bartuma, Anna Ebbesson, Sofia Westbom-Fremer, Anna Måsbäck, Susanne Malander, Mef Nilbert, Ingrid A. Hedenfalk

**Affiliations:** ^1^Division of Oncology and Pathology, Department of Clinical Sciences, Skåne University Hospital, Lund University, Lund, Sweden; ^2^CREATE Health Strategic Center for Translational Cancer Research, Lund University, Lund, Sweden; ^3^Department of Clinical Pathology, Division of Laboratory Medicine, Skåne University Hospital, Lund, Sweden; ^4^Clinical Research Centre, Hvidovre University Hospital, Copenhagen University, Hvidovre, Denmark

**Keywords:** clear cell ovarian cancer, gene expression profiling, deep sequencing, inter-tumor heterogeneity, chromatin modification, Rho GTPase, *ERBB2*

## Abstract

**Objective:**

Ovarian clear cell carcinomas (OCCCs) constitute a rare ovarian cancer subtype with distinct clinical features, but may nonetheless be difficult to distinguish morphologically from other subtypes. There is limited knowledge of genetic events driving OCCC tumorigenesis beyond *ARID1A*, which is reportedly mutated in 30–50% of OCCCs. We aimed to further characterize OCCCs by combined global transcriptional profiling and targeted deep sequencing of a panel of well-established cancer genes. Increased knowledge of OCCC-specific genetic aberrations may help in guiding development of targeted treatments and ultimately improve patient outcome.

**Methods:**

Gene expression profiling of formalin-fixed, paraffin-embedded (FFPE) tissue from a cohort of the major ovarian cancer subtypes (cohort 1; *n* = 67) was performed using whole-genome cDNA-mediated Annealing, Selection, extension and Ligation (WG-DASL) bead arrays, followed by pathway, gene module score, and gene ontology analyses, respectively. A second FFPE cohort of 10 primary OCCCs was analyzed by targeted DNA sequencing of a panel of 60 cancer-related genes (cohort 2). Non-synonymous and non-sense variants affecting single-nucleotide variations and insertions or deletions were further analyzed. A tissue microarray of 43 OCCCs (cohort 3) was used for validation by immunohistochemistry and chromogenic *in situ* hybridization.

**Results:**

Gene expression analyses revealed a distinct OCCC profile compared to other histological subtypes, with, e.g., *ERBB2, TFAP2A*, and genes related to cytoskeletal actin regulation being overexpressed in OCCC. ERBB2 was, however, not overexpressed on the protein level and *ERBB2* amplification was rare in the validation cohort. Targeted deep sequencing revealed non-synonymous variants or insertions/deletions in 11/60 cancer-related genes. Genes involved in chromatin remodeling, including *ARID1A, SPOP*, and *KMT2D* were frequently mutated across OCCC tumors.

**Conclusion:**

OCCCs appear genetically heterogeneous, but harbor frequent alterations in chromatin remodeling genes. Overexpression of *TFAP2A* and *ERBB2* was observed on the mRNA level in relation to other ovarian cancer subtypes. However, overexpression of *ERBB2* was not reflected by HER2 amplification or protein overexpression in the OCCC validation cohort. In addition, Rho GTPase-dependent actin organization may also play a role in OCCC pathogenesis and warrants further investigation. The distinct biological features of OCCC discovered here may provide a basis for novel targeted treatment strategies.

## Introduction

Ovarian clear cell carcinomas (OCCCs) represent a distinct histological and clinical subset accounting for 5–10% of epithelial ovarian cancers (EOCs), but among Asian women, the rate is at least 15%. In general, EOC is characterized by an adverse prognosis, with a relative 5-year survival of <50% (1). The survival of EOC is influenced by stage, age at diagnosis, histological grade, residual tumor burden after surgery, and, not least, histopathological subtype (2–4). Of the four major histopathological subtypes [serous, endometrioid (EM), mucinous (MUC), and clear cell ovarian cancers], OCCCs stand out as a group with distinct clinical characteristics (5). They are considered type 1 tumors, arising gradually through progression from benign precursors associated with endometriosis, and eventually developing into invasive carcinomas (6, 7). Although often diagnosed in early stages, and with a younger median age at diagnosis compared to, e.g., high-grade serous ovarian carcinomas, stage III–IV OCCC confers an inferior prognosis relative to stage-matched serous tumors (8, 9). The outcome for patients with OCCC, regardless of stage, is also worse than for patients with EM ovarian cancer (10). The inferior prognosis may be related to a high prevalence of primary chemotherapy resistance, primarily to platinum-based drugs. This may be due to both a low proliferation rate (a prototypic type 1 tumor feature) and intrinsic resistance mechanisms such as expression of apoptosis regulators, e.g., p53 and p21, although this is not entirely clear [(11), reviewed in Ref. (12)].

Regardless of their relatively distinct clinical features, OCCCs may be difficult to distinguish histopathologically from other epithelial ovarian tumors, especially of the EM and high-grade serous subtypes. Immunohistochemical (IHC) markers such as Napsin A and HNF1β are useful tools; nevertheless, the diagnosis may sometimes be difficult to confirm using IHC alone (13, 14). Using global gene expression analysis, however, distinct transcriptional signatures of OCCC have been described, capturing not only the morphological appearance but also the biological behavior of this ovarian cancer subtype (15, 16). Furthermore, *ARID1A*, involved in chromatin remodeling, has been reported to be mutated in a subset of OCCCs, and other components of the chromatin remodeling system have also recently been reported to be affected in EM and high-grade serous ovarian cancer [(17–19), reviewed in Ref. (20)]. Table 1 summarizes the most common aberrations reported in OCCC.

**Table 1:** **Common alterations in ovarian clear cell carcinoma**.

Genomic alterations	Altered in	Reference
ARID1A (loss of function)	~50%	(18)
PIK3CA (gain of function)	~50%	(21, 22)
TP53 (loss of function)	~25%	(21)
KRAS (loss of function)	~10%	(21, 23)
cMET (gain of function)	<5%	(21)
PTEN (loss of function)	<5%	(21)
**mRNA expression**		
FXYD2 (upregulated)	NA	(24)
WT1 (downregulated)	NA	(24)
TFAP2A (upregulated)	NA	(25)
ESR1 (downregulated)	NA	(25)
**Protein expression**		
Napsin A (amplification)	~80%	(13)
HNF-1B (amplification)	>80%	(26)
AKT (amplification)	~70%	(27)
PTEN (loss)	~50%	(21)
cMET (loss)	~25%	(21)
HER2 (amplification)	~12%	(28)

In the present study, we aimed to further characterize OCCCs and their histotype-specific genetic aberrations using global gene expression profiling as well as targeted deep sequencing of a set of well-established cancer genes. Increased knowledge of genetic aberrations in OCCCs may help to guide future development of targeted treatments and clinical trials.

## Materials and Methods

### Tumor Material

The tumor material used in the present study was retrieved from three different cohorts and is referred to throughout as cohorts 1, 2, and 3. *Cohort 1* comprises 72 ovarian carcinomas of mixed histopathologic subtypes, partly outlined in Jönsson et al. (29). The tumors in cohort 1 were collected among Swedish and Danish Lynch syndrome carriers (*n* = 28) and Swedish sporadic cases (*n* = 44). Formalin-fixed, paraffin-embedded (FFPE) tissue was available from all tumors. *Cohort 2* consists of 11 OCCCs, 9 of which were obtained from the Skåne University Hospital (Sweden) ovarian tumor biobank, and 2 of which overlapped with cohort 1. FFPE tissue was available from all tumors. *Cohort 3* consists of 43 OCCCs from Skåne University Hospital arranged in a tissue microarray (TMA). Clinicopathological data for all cohorts are summarized in Table 2. All tumor samples were collected at primary surgery, and the patients had not received prior chemotherapy. Histopathological subtype and grade were determined according to Silverberg and the WHO 2003 classification, which was used at the time of diagnosis (30, 31). For tumors in cohorts 2 and 3, the histopathological subtype was determined according to the more recent WHO 2014 classification (32). All tumors were staged according to the International Federation of Gynecology and Obstetrics criteria (33). Ethical approval for the study was granted from the ethics committee in Region Hovedstaden, Denmark, and from the Lund University ethics committee, Sweden, and all patients provided written, informed consent.

**Table 2:** **Clinical characteristics of ovarian clear cell carcinomas (OCCCs) in the study cohorts**.

	Cohort 1		Cohort 2	Cohort 3
		
	All tumors	OCCC	OCCC	OCCC
		
	*n* = 67	*n* = 15	*n* = 10	*n* = 43
**Age at diagnosis (years)**				
Median	51	48	66.5	63
Range	27–78	34–60	51–90	41–90
**Stage, ***n*** (%)**				
I	28 (46)	13 (92)	7 (78)	27 (63)
II	9 (15)	0	1 (11)	6 (14)
III	20 (33)	1 (8)	1 (11)	9 (21)
IV	4 (7)	0	0	1 (2)
Missing	6	1	1	0
**Age of formalin-fixed, paraffin-embedded blocks (years)**				
Median	12	19	4.5	8
Range	3–54	3–38	2–16	8–13
**Histology, ***n*** (%)**				
Clear cell	15 (22)	15 (100)	10 (100)	43 (100)
Serous	31 (46)	–	–	–
Endometrioid	18 (27)	–	–	–
Mucinous	3 (4)	–	–	–

### RNA Extraction and Gene Expression Analysis

RNA was extracted from all FFPE samples in cohort 1 (*n* = 72 ovarian carcinomas). As outlined previously, non-necrotic tumor areas with >70% tumor cell content were selected from three to five 10 µm sections of Hematoxylin and Eosin stained tissue per tumor and RNA was extracted from these areas using the High Pure RNA Paraffin Kit (Roche, Castle Hill, Australia) (29). RNA concentrations were determined using a NanoDrop Spectrophotometer (NanoDrop Technologies, Wilmington, DE, USA); 68/72 tumors met the quality criteria (300 ng of RNA with a 260/280 ratio >1.8). Gene expression analyses were performed at the SCIBLU Genomics Centre, Lund University, Sweden. The cDNA-mediated Annealing, Selection, extension and Ligation (WG-DASL) assay (Illumina Inc., San Diego, CA, USA), containing 24,526 probes representing 18,626 unique genes, was used for whole-genome expression analysis. The samples were randomized on the chips and were profiled following the manufacturer’s instructions. BeadChips were then scanned on a BeadArray™ Reader using BeadScan software (v4.2), during which fluorescence intensities were read and images extracted. A raw average signal intensity >250 for each probe and >8,000 detected genes/sample were required for further analysis; 67/68 samples met these criteria. The raw data are freely available in NCBI’s Gene Expression Omnibus (34) through GEO series accession number GSE37394. The raw data were quantile normalized, log2 transformed and subjected to a presence filter of 80% across probes, with a detection *P*-value ≤0.01, leaving 12,747 probes. Biological duplicates were averaged and when multiple probes identified the same gene, the probe with the highest variance across tumors was chosen to represent the gene, resulting in 10,000 remaining probes. The data were then mean centered across samples using R version 3.2.2 (35). Thereafter, the data were imported into the MeV version 4.9.0 software (36) and a multiclass significance analysis of microarrays (SAM) analysis (37) using the histological subtypes as groups (serous, EM, MUC, and clear cell) was performed. A SAM analysis, using 1,000 random permutations, was used to identify the 5% most significant differentially expressed genes between subgroups, with a false discovery rate (FDR) <5%. OCCCs were compared to the other subtypes individually using the same settings. Pearson correlation was used for distance metric selection and complete linkage was used for cluster generation. Enrichments of gene ontology (GO) terms were explored using the online Panther gene list analysis tool with default settings for *Homo sapiens*, using a binomial statistical overrepresentation test and Bonferroni correction for multiple testing with the 10,000 genes as background (38). GO terms were compared using the online Gene Semantic Similarity Analysis and Measurement tool (G-SESAME) with default settings (39), generating a semantic similarity score between GO terms of 0 (low similarity) to 1 (high similarity). Gene set analysis using gene sets from the REACTOME database [1,764 pathways, ConsensusPathD (40)] was performed using the GS-Reg package version 1.8 in R with 1,000 permutations, a minimum gene count of 7 and a *P*-value cutoff of 0.001 for all genes (41). Genes from the SAM analysis were analyzed using the ConsensusPathDB, with a minimum gene count of 7 and a *q*-value cutoff of 0.05 for REACTOME pathways. Venn diagrams were generated using the online Venn Diagram tool from the Bioinformatics and Evolutionary Genomics web page.^1^ Gene module score comparisons according to gene modules defined by Desmedt et al. were performed as previously described (42, 43). Statistical tests (Kruskal–Wallace and Dunn’s test) and boxplots were performed using R version 3.2.2.

### DNA Extraction and Targeted Sequencing

DNA was extracted from all FFPE samples in cohort 2 (*n* = 11 OCCCs) using the AllPrep DNA/RNA FFPE Kit (Qiagen, Venlo, Netherlands). Two 10 µm tissue sections/tumor were used for DNA extraction and >1 μg DNA was obtained from all samples. Three micrometer hematoxylin stained sections, retrieved immediately before the sections used for DNA extraction, were reviewed by a gynecological pathologist (SWF) to establish tumor cellularity, which ranged from 65 to 99%, with a median of 90% (data not shown). DNA concentrations were measured using the Qubit Fluorometric Quantitation^®^ (Life Technologies, Thermo Fisher Scientific, Waltham, MA, USA), and a qPCR-based DNA quality control was performed using the Trusight Tumor Sample Preparation Kit (Illumina Inc.), with ΔCT values <6.5 considered sufficient. One sample was excluded due to poor DNA quality. For the remaining 10 samples, 600 ng genomic DNA/sample (50 ng/µl) was used for targeted DNA sequencing. Sample preparation and sequencing was performed at Oxford Gene Technology™ (OGT, Oxford, Great Britain). The Next-Generation Sequencing SureSeq™ solid tumor panel, a hybridization-based enrichment tool targeting all codons of the exons in the included genes, was used. The gene panel consists of 60 key cancer genes and has been validated for research use on FFPE samples^2^ (Table S1 in Supplementary Material).

Alignment was performed using the human build GRCh37 reference genome and Burrows–Wheeler Aligner (version 0.7.10). Local realignment was carried out using the Genome Analysis Tool Kit (GATK, version 1.6), and duplicate reads were removed using Picard version 1.107. Variant calling was performed using the BCbio-nextgen tumor only prioritization pipeline^3^ with Mutect2 from GATK as variant caller and default settings (44). Variants were annotated using the Variant Effect Predictor (45). Variants were classified using the GEnome MINIng (GEMINI) framework to filter out possible germline variants (46), with a variant allele frequency filter cutoff of >5%.

### IHC and Chromogenic *In Situ* Hybridization (CISH)

Immunohistochemical and CISH evaluation of *ERBB2* were performed using standard procedures for HER2 testing in breast cancer. The anti-HER2/neu rabbit monoclonal antibody 4B5 (Ventana, Tucson, AZ, USA) was used for IHC and the 2,4-dinitrophenyl (DNP)-labeled HER2 probe and digoxigenin (DIG)-labeled Chr17 were used for CISH. The HER2 gene and Chr17 signals were detected using the ultraView SISH DNP and ultraView Red ISH DIG detection kits (Ventana). Tumors with an IHC score of 3+ and/or a gene/centromere ratio ≥2 were considered positive (Figure S1 in Supplementary Material). Anti-AKT-pS473 (Dako, Denmark) staining was performed using Dako EnVision™ System/HRP. A 1:10 dilution was used with Dako Retrieval Solution (pH 9). Any staining was considered positive.

## Results

### Gene Expression Analyses Reveal Distinct OCCC Profiles and Involvement of Rho GTPases

**Figure 1:**
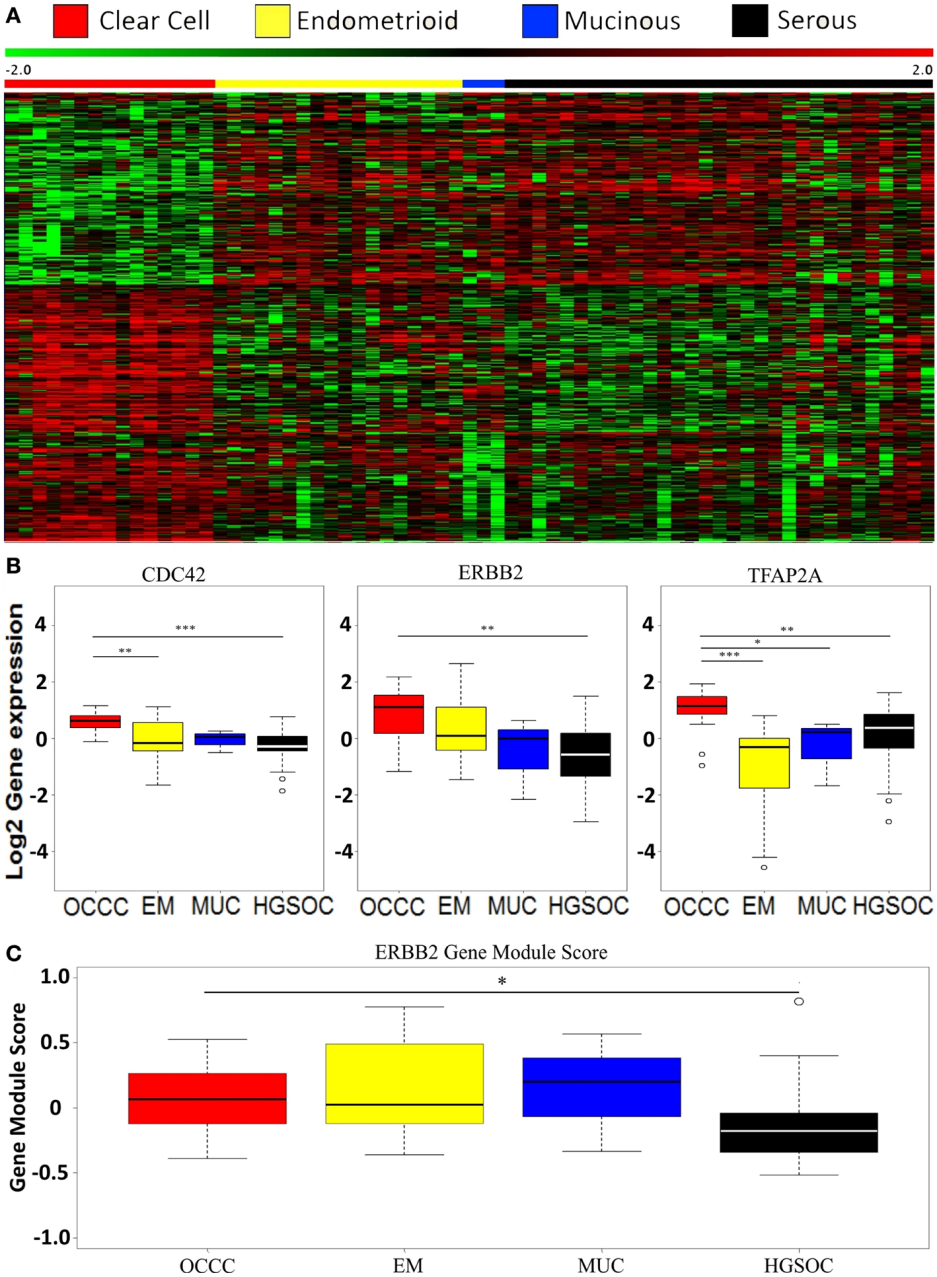
**Global gene expression profiles in ovarian cancer**. **(A)** Heatmap of the 505 most significant differentially expressed genes based on a supervised multiclass significance analysis of microarrays analysis comparing the four histopathological subtypes (serous, EM, MUC, and clear cell) in cohort 1. **(B)** Boxplots illustrating differential expression of *CDC42, ERBB2*, and *TFAP2A* between the histological subtypes using Dunn’s test for difference. *P < 0.05; **P < 0.01; ***P < 0.001. **(C)** Boxplot illustrating *ERBB2* gene module scores in the histological subtypes as defined by Desmedt et al. using Dunn’s test for difference. *P < 0.05. OCCC, ovarian clear cell carcinoma; EM, endometrioid; MUC: mucinous; HGSOC: high-grade serous ovarian cancer.

We wanted to investigate the gene expression profiles of OCCC compared to the other ovarian cancer subtypes. In a previous study of Lynch syndrome-associated ovarian cancer, we reported a distinct gene expression pattern of OCCC compared to other subtypes using unsupervised hierarchical clustering. This was found to be irrespective of Lynch status (29). Hence, we performed a supervised multiclass SAM analysis without a variance filter using all 10,000 genes and histological subtypes as groups, thereby identifying 505 differentially expressed genes (FDR < 5%). OCCCs were found to display a distinct transcriptional pattern compared to all the other histological subtypes (Figure 1A). The pathway analysis revealed four altered pathways (Table 3), with the extracellular matrix (ECM) organization, axon guidance, and developmental biology pathways exhibiting many genes in common. We next applied a variance filter to select the 2,500 genes with the highest variance across tumors. This was done to select as many targets as possible throughout the signaling network hierarchy, as proposed by Komurov and Ram (47). This SAM analysis identified 504 significant genes (FDR < 10%), and the subsequent pathway enrichment analysis revealed genes belonging to both the NCAM signaling and TFAP2A transcription pathways, as well as pathways involved in MAPK signaling. In addition, pathways connected to ECM organization, axon guidance, and developmental biology were also differentially expressed (Table 3). Although three pathways were found in common between the two SAM analyses, only 48% (271/504) of the genes overlapped (data not shown).

**Table 3:** **Pathway enrichment of differentially expressed genes identified using significance analysis of microarrays analysis with/without a variance filter**.

Pathway^a^	*q*-Value
**No variance filter**	
Extracellular matrix (ECM) organization	0.026
Axon guidance	0.045
Developmental biology	0.045
Transport of glucose and other sugars, bile salts and organic acids, metal ions and amine compounds	0.045
**Variance filter**	
NCAM signaling for neurite out-growth	0.012
NCAM1 interactions	0.015
Transcriptional regulation by the AP-2 (TFAP2) family of transcription factors	0.017
ECM organization	0.017
ECM proteoglycans	0.024
GPCR ligand binding	0.039
Axon guidance	0.039
Signaling by GPCR	0.039
RAF/MAP kinase cascade	0.039
SHC1 events in EGFR signaling	0.039
SOS-mediated signaling	0.039
GRB2 events in EGFR signaling	0.039
Gastrin-CREB signaling pathway *via* PKC and MAPK	0.039
Signaling to p38 *via* RIT and RIN	0.039
ARMS-mediated activation	0.039
MAPK family signaling cascades	0.039
Frs2-mediated activation	0.039
MAPK1/MAPK3 signaling	0.039
Prolonged ERK activation events	0.043
Signaling by Leptin	0.045
Signaling to RAS	0.045
Developmental biology	0.045
Interleukin receptor SHC signaling	0.047
VEGFR2-mediated cell proliferation	0.049

*^a^Minimum gene presence: 7; false discovery rate <0.05*.

To further explore this finding, we performed a gene set analysis of the whole dataset (10,000 genes), revealing genes of the axon guidance pathway to be differentially expressed between OCCC and the other subtypes combined (*P* < 0.001, 156/251 genes in pathway present in dataset). However, when OCCCs were compared to the other subtypes individually, the genes of the axon guidance pathway were only found to be differentially expressed between the OCCC and the EM subtypes (*P* < 0.001; File S1 in Supplementary Material).

**Figure 2:**
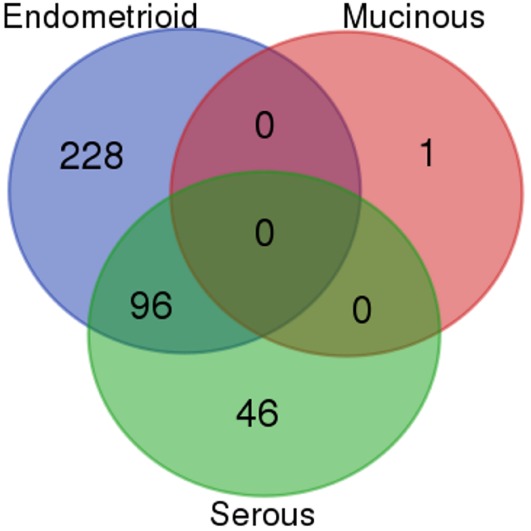
**Venn diagram of the overlap of differentially expressed Reactome pathways between ovarian cancer subtypes**. The number of overlapping Reactome pathways from the gene set analysis of ovarian clear cell carcinoma compared to the high-grade serous, mucinous, and endometrioid subtypes, respectively.

Comparing the individual differentially expressed pathways between OCCC and the other subtypes, we found the highest number of differentially expressed pathways when comparing OCCC to the EM subtype. A total of 324 pathways, encompassing 4,599 genes, included genes that were differentially expressed between the OCCC and EM subtypes (Figure 2). However, a significant overlap of genes between 295 of these pathways (91%) was observed. Furthermore, several pathways were found to be general and were probably a result of these pathways containing other smaller pathways, resulting in an elevated number of significant pathways. However, 30% (96/324) of these pathways had genes which were differentially expressed between OCCC and the serous and EM subtypes combined. Among these 96 pathways were the “TFAP2 (AP-2) family regulates transcription of growth factors and their receptors” pathway, the “Chromatin organization” pathway, and several PI3-kinase and AKT associated pathways (File S1 and File S2 in Supplementary Material). A comparison of the genes in the differentially expressed pathways revealed three genes of interest: the Rho GTPase *CDC42*, the growth factor receptor *ERBB2*, and the transcription factor *TFAP2A*. These genes were differentially expressed between subtypes, with overexpression in OCCC (Figure 1B), and were also present in many of the altered pathways in both the SAM and whole gene set analyses. Genes in the Rho GTPase pathways were also differentially expressed between OCCC and both the EM and serous subtypes; however, no direct overlap in pathways between subtypes was observed.

The overexpression of *ERBB2* in the OCCC subtype prompted us to investigate the protein expression and copy number status of *ERBB2* using a TMA. Only one of 43 OCCC tumors (2%) showed overexpression and amplification of ERBB2 (Figure S1 in Supplementary Material), and one tumor displayed amplification (ratio ≥ 2) with weak protein expression (1+). Three tumors (7%) displayed weak protein expression (1+) without amplification. This suggests that while *ERBB2* may be overexpressed in OCCC on the mRNA level relative to other histological subtypes of ovarian cancer, this does not result in overexpression on the protein level, nor does it appear to be caused by amplification of the *ERBB2* gene. Further support of this was found when applying a module score for the ERBB2 pathway from breast cancer to our data (*P* = 0.2896, two sided *t*-test, 17/28 genes present) (43), which did not provide evidence for ERBB2 mediated activity in OCCC (Figure 1C). However, there was a significant difference between the OCCC and serous subtypes (*P* = 0.0147, Dunn’s test, 17/28 genes present), with a lower module score in the serous subtype, in line with a previous study from our group (42).

In a previous study, we evaluated the protein expression of mTOR, PTEN, and EGFR in OCCCs in cohort 1 (*n* = 12). EGFR was expressed in two tumors (17%), PTEN in four tumors (33%), and mTOR in 7 tumors (58%) (29). pAKT was evaluated using the previously mentioned TMA of 43 OCCCs. pAKT protein expression was observed in 23 tumors (58%).

Next, we performed a GO term similarity score analysis using the genes from the SAM analysis without variance filter and compared those to a previous study of OCCC (including the clear cell, serous and EM subtypes) (16) using G-SESAME. The similarity score was 0.683, confirming our findings, and GO terms above this threshold were related to anatomical structure development, cell differentiation, and signaling (Table S2 in Supplementary Material).

Taken together, these findings indicate that different mechanisms involved in structural modifications, such as Rho GTPases and chromatin organization genes, may play an important role in tumor development and progression in OCCC. In addition, upregulation of the transcription factor *TFAP2A* may be involved in the distinct transcriptional differences observed between OCCC and other subtypes. Furthermore, differential activation of the PI3K and mTOR pathways, implicated in the development of type I ovarian carcinomas, between OCCC and both EM and serous tumors, may indicate deregulation of the PI3K/mTOR/AKT pathway in OCCC.

### Targeted Deep Sequencing Uncovers Variants in Genes Involved in Chromatin Remodeling and Transcriptional Regulation in OCCC

The transcriptional differences observed across the ovarian cancer subtypes, specifically the appearance of a distinct OCCC gene expression profile, prompted further analyses of the potential underlying mutational drivers of OCCC. We therefore performed targeted deep sequencing of 60 cancer-related genes in the 10 OCCCs from cohort 2; >99% of the target bases had at least 30X coverage across all 10 samples, with a mean target coverage of 534X (range 314X-698X). A total of 8,595 variants were called, spanning 3,849 unique variants across the 10 tumors. Of these, 1,244 were tagged by GEMINI as potential germline variants, while Mutect2 filtered out 7,319 variants, leaving 32 variants (0.3%) using a minor allele frequency cutoff of 5%.

**Figure 3:**
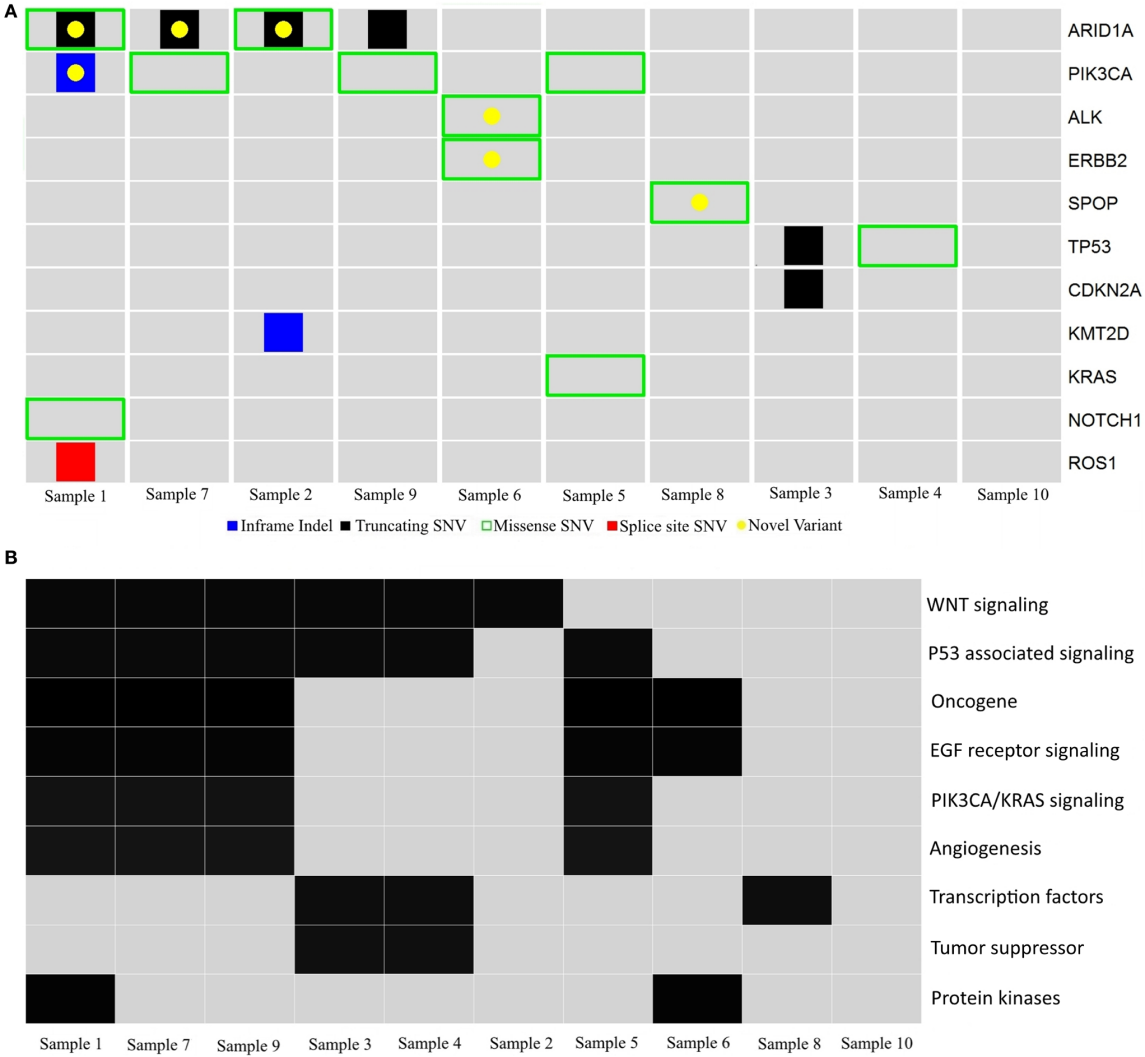
**Schematic diagram of gene variants in ovarian clear cell carcinoma**. **(A)** Illustration of probable somatic variants in genes (rows) across tumors (columns) in cohort 2. Red, splice site SNV; black, truncating SNV; green, missense SNV; blue, inframe indels; yellow, unreported variants. **(B)** Summary of pathways and gene families (rows) affected by probable somatic variants across tumors (columns) in cohort 2.

The 32 mutations were manually compared to the COSMIC, ExAC, and ENSEMBL (dbSNP148) databases to identify previously reported variants and filter out variants with a global minor allele frequency >0.0001 (i.e., 1/10,000, suspected to be germline SNPs). Twenty probable somatic variants were identified in 11 genes, with a variant range from 0 to 5 (median 2), supporting the notion of a genomically stable subtype. Two variants were inframe indels, 10 were missense variants and eight were truncating variants. Forty-five percent (9/20) of the variants have not been previously reported (Figure 3A and Table 4). Using the gene family nomenclature provided by the online Molecular Signatures Database (MSigDB V5.1; Broad Institute, MA), 2 of these genes are tumor suppressors (*CDKN2A, TP53*), 6 are oncogenes (*ERBB2, PIK3CA, KRAS, ALK, NOTCH1, ROS1*), 2 are transcription factors (*TP53, SPOP*), and 3 are protein kinases (*ERBB2, ALK, ROS1*). Next, the PantherDB online tool was used to explore potential affected pathways. Several pathways, including the TP53, WNT, EGF, and PIK3CA/KRAS pathways were found to be affected (Figure 3B).

**Table 4:** **Somatic variants in ovarian clear cell carcinoma and their reported RsID and status in the COSMIC database**.

	Gene	Alteration	RsID	COSMIC
Sample 1	ARID1A	Gln1614*	NR	NR
Sample 1	NOTCH1	N280S	rs367825691	NR
Sample 1	PIK3CA	EDLLNPI453del	NR	NR
Sample 1	ROS1	V1881_splice	rs772648589	NR
Sample 2	ARID1A	P476*	NR	NR
Sample 2	ARID1A	Leu1054Phe	NR	NR
Sample 2	KMT2D	Gln3745dup	rs775492040	NR
Sample 3	CDKN2A^a^	Glu69*	rs121913383	COSM13281
Sample 3	TP53^a^	Asn268*	NR	COSM6583
Sample 4	TP53	Arg181Cys	rs587782596	COSM11090
Sample 5	KRAS	Gly12Val	rs121913529	COSM520
Sample 5	PIK3CA	His1047Arg	rs121913279	COSM775
Sample 6	ALK	Gly926Arg	NR	NR
Sample 6	ERBB2	Ile989Met	NR	NR
Sample 7	ARID1A^a^	Tyr1055*	NR	NR
Sample 7	PIK3CA^a^	Lys111Glu	NR	COSM13570
Sample 8	SPOP	Lys115Glu	NR	NR
Sample 9	ARID1A	Gln537*	NR	NR
Sample 9	ARID1A	Gln2176*	NR	COSM1582021
Sample 9	PIK3CA	His1047Arg	rs121913279	COSM775

NR, not reported; *, truncating variant; splice, splice site variant; del, deletion.

*^a^Validated using available exome sequencing data*.

*ARID1A* and *PIK3CA* variants, previously identified in OCCC, were present in 4/10 (40%) tumors, and co-occurred in three tumors (30%). Samples 2 and 9 had >1 *ARID1A* variant each. The six *ARID1A* variants included three small indels and three missense variants; five of these variants resulted in protein truncation downstream of the variation site (Table 4). Only one variant, the truncating variant Gln2176 in Sample 9, has previously been reported, although not in ovarian cancer. Of the three *PIK3CA* variants, the missense variant His1047Arg occurred in two samples and has been associated with ovarian cancer in 91 tumors in the COSMIC database. Two variants had previously been reported in COSMIC, while the EDLLNPI453del variant in Sample 1 has not been reported.

The *ERBB2* variant (p.989I > M) has not previously been reported in any of the databases used in this study. However, it is located outside the functional domains of the *ERBB2* gene, and the significance of this finding is therefore unknown (data not shown).

Two *TP53* single nucleotide deletion variants were found, both of which are reported in COSMIC (Table 4). The missense variant Arg181Cys has been identified in breast and endometrial cancer according to COSMIC, but not in ovarian cancer.

The *KRAS* variant found in one tumor (c.35G > T) has previously been reported as a somatic variant in ovarian cancer (COSM520).

The *CDKN2A* variant has previously been identified in COSMIC, however not in ovarian cancer. Finally, variants in the genes *ROS1, NOTCH1, ALK, KMT2D*, and *SPOP* have to our knowledge not previously been reported in ovarian cancer.

In summary, genes previously reported to be mutated in OCCC, including *ARID1A, PIK3CA*, and *ERBB2*, were identified in the present cohort. Variants in samples 3 and 7 were validated using available exome data with paired tumor and blood, supporting the use of FFPE tissue for confident variant calling using strict criteria as implemented in the BCbio pipeline (Table 4). Overall, genes affected by potential somatic variants in OCCCs in the present study included *KMT2D, KRAS, ARID1A, PIK3CA, TP53, ERBB2, NOTCH1*, and *SPOP*. These genes, spanning variants over 8/10 tumors, are associated with a comprehensive list of functions that relate to multiple cancer driving cellular processes. However, in common are chromatin remodeling and transcriptional regulation relying on chromatin, providing a possible explanation to a common underlying cause in OCCC pathogenesis.

## Discussion

Differences in the clinical behavior between the histopathological subtypes of ovarian cancer are well known, and differences have also been reported on the molecular level (16, 29–31), with OCCCs displaying remarkably distinct features including resistance to chemotherapy and distinct gene expression profiles compared to other ovarian cancer subtypes (9–11). In the present study, we investigated transcriptional and mutational landscapes in order to shed light on the underlying molecular features of these cancers.

Gene expression profiling clearly distinguished OCCCs as a unique subtype compared to other subtypes of ovarian cancer. A GO analysis comparing the genes in the present study and the OCCC study by Zorn et al. (16) revealed a similarity of GO terms linked to regulation of various biological processes often found to be deregulated in cancer (48). Interestingly, the enrichment test revealed overrepresentation of the “ECM organization,” “axon guidance,” and “developmental biology” pathways in the four-way SAM analysis comparing all four histological subtypes in our study. A common denominator for these pathways is the Rho family of GTPases. Rho GTPases are small GTP binding proteins highly conserved through species. They regulate a broad variety of cellular functions, including actin polymerization/organization and cell migration, and are frequently aberrantly expressed in cancer (49). *CDC42* and *RhoB*, both members of this family, were significantly differentially expressed in the present study. Interestingly, a study by Canet et al. suggested that Rho GTPases may contribute to the distinct morphology of OCCC. They found lower *CDC42* mRNA in OCCCs compared to the high-grade serous subtype (50), although our observations indicated the converse—higher *CDC42* levels in OCCC than in the EM and high-grade serous subtypes. The discrepancy may be due to differences in stage between the two studies, with the majority of tumors in the present study being stage I, whereas the tumors in the study by Canet et al. were primarily stage III and IV (8, 9). However, our findings correlate well with *CDC42* and *RhoB* expression levels found in the Ovarian cancer database of Cancer Science Institute Singapore (CSIOVDB), reporting significantly higher *CDC42* and *RhoB* levels in OCCC compared to the other subtypes (51).

Through gene expression analysis, we observed differences in expression of genes belonging to the PI3K/mTOR/AKT pathway and this was supported by IHC stainings, which showed loss of PTEN and expression of both mTOR and pAKT in OCCC tumors. This is in line with a recent study of 521 OCCCs, which reported that the PI3K/mTOR/AKT pathway was altered in >50% of the tumors and suggested significant cross talk with other pathways, such as the RAS/RAF/MEK/ERK pathway (21), which was found to be differentially expressed in our study (Table 3). In support of this, protein phosphatase 2A (PP2A) has recently been suggested as a potential target for the cross talk between these two pathways (52). Taken together, with PP2A acting upstream of the actin regulating RhoA/ROCK/MLC pathway (53), these findings suggest a mechanism whereby Rho GTPases connect these pathways, possibly thereby contributing to the pathogenesis of OCCC. Using a gene set-based integrative approach, Chang et al. recently reported deregulation of pathways related to the PI3K cascade in OCCC compared to normal control tissue. They also described deregulation of the GO term “Rho guanyl nucleotide exchange factor activity,” also in line with our findings (54). The gene set analysis performed here confirmed these results, as pathways related to PI3K/mTOR/AKT signaling, implicated in the development of type I carcinomas including OCCC, were also deregulated between OCCC and both the EM and serous subtypes in our data set.

*ERBB2* has been proposed to constitute a viable therapeutic target in OCCC based on amplification of *ERBB2* with concomitant protein overexpression in 14% of OCCC tumors in one study (55). However, although *ERBB2* was upregulated compared to other subtypes in our cohort, downstream effectors of the pathway appeared unaffected, and overexpression was not observed on the protein level, suggesting that the signaling pathway may not be activated in OCCC. These findings are supported by others who have investigated HER2 expression in ovarian cancer and found no prognostic value (56, 57). These studies are however limited due to smallnumbers. The lack of concordance and prognostic value may however be due to the apparent inactivity of the HER2 pathway reported in the present study. The cause of this remains unclear, but incomplete amplification of the *HER2* region on chromosome 17 has been reported in a subset of *HER2*-amplified breast cancers in the absence of protein overexpression (58). In summary, our findings confirm that the PI3K/mTOR/AKT pathway may play an important role in OCCC pathogenesis; however, our data also implicate Rho GTPases in this context, possibly as a mediator between several pathways involved in the development of OCCC.

Transcription factor AP2-α *(TFAP2A)* is a tumor suppressor gene acting through *TP53* in breast cancer, and its expression is known to inhibit cell division (59). Higher mRNA levels of *TFAP2A* were found in OCCC compared to the three other subtypes, which is supported by previous findings (25). Furthermore, increased *TFAP2A* mRNA levels have been reported as a positive prognostic marker for overall survival in EOCs of all subtypes (60). Of interest, *ERBB2* expression and pathway activation has been linked to *TFAP2A* expression, and the “Transcriptional regulation by the AP-2 (TFAP2) family of transcription factors” pathway, which includes *ERBB2*, was found to be differentially expressed in our study. However, the underlying mechanism has yet to be determined.

To extend our findings, we applied targeted next-generation sequencing using the SureSeq™ solid tumor panel to a cohort of 10 OCCCs. The variant pattern revealed a heterogeneous mutational landscape, but is largely in line with what has previously been reported. The oncogene *KRAS* is often mutated in low proliferating, type 1 ovarian tumors (23). A single *KRAS* variant was found in our cohort, in line with the previously reported overall variant frequency of 7–14% in OCCC (21, 23). Altered chromatin remodeling has been reported as a driver of tumorigenesis in OCCC, with *ARID1A* being involved in early stages in conjunction with *PIK3CA* (61). Variants in *ARID1A* and *PIK3CA* were collectively observed in five tumors, consistent with previous reports stating variant frequencies of 30–50% (18). Interestingly, in a mutant mouse model of *ARID1A* dependent OCCC, concurrent activation of *PIK3CA* was required for tumor formation, suggesting that the SWI/SNF chromatin remodeling and PI3K pathways converge in the development of OCCC (19). Other PI3K pathway aberrations, such as loss of *PTEN*, may contribute to OCCC pathogenesis in the absence of *PIK3CA* activation.

Intriguingly, two other genes involved in chromatin remodeling, *KMT2D* and *SPOP*, were identified in the present study, suggesting that although *ARID1A* has been the focus of attention, other defects in the chromatin remodeling system may contribute to OCCC tumorigenesis. Importantly, mutations in the three genes were mutually exclusive. *SPOP* indirectly affects heterochromatin maintenance through ubiquitination of the DAXX protein, thereby contributing to, e.g., renal clear cell tumorigenesis (62). *KMT2D*, on the other hand, is part of the COMPASS-like complex (COMplex of Proteins ASsociated with Set1) responsible for mono-methylation of H3K4, and is recruited by, e.g., nuclear binding receptors such as the estrogen receptor and by other transcription factors such as p53, which may be significant for OCCC tumorigenesis (63).

Of the 20 potential somatic variants identified across tumors in the current study, 8 were previously reported as being somatic, while the remaining variants were considered to be potential somatic variants. The potential tumorigenic effect of these 20 variants is unclear and requires further investigation.

In conclusion, overexpression of both *ERBB2* and *TFAP2A* is frequent in OCCC, but in the case of *ERBB2*, does not translate to protein overexpression. The role of *TFAP2A* in cancer progression appears to vary according to cancer type and remains to be determined in OCCC development. Deregulation of Rho GTPases was common among OCCCs compared to the other subtypes of ovarian cancer. Rho GTPases have been the focus of attention for their role in tumorigenesis, especially in invasiveness and metastasis development. Several inhibitors targeting effectors and activators of the Rho GTPases are available, and their potential role in OCCC remains to be explored. Taken together, the complex networks in which Rho GTPases participate seem to play a critical role in OCCC pathogenesis, potentially in conjunction with the PI3K/mTOR/AKT pathway. On both the genomic and gene expression levels, chromatin remodeling and subsequent deregulation of gene expression may also play a critical role in the development and progression of OCCC. Variants in *KMT2D, ARID1A*, and *SPOP* reflect the diversity of genomic variations in OCCC. These genes play a critical role in regulating gene expression and silencing, and the variation pattern detected may contribute to the observed resistance to chemotherapy. Further investigations are needed to elucidate the possible interplay between Rho GTPases and the PI3K/mTOR/AKT pathway, as well as the role of chromatin remodeling in OCCC pathogenesis.

## Ethics Statement

This study was carried out in accordance with the recommendations of the ethics committee in Region Hovedstaden (Denmark) and from the Lund University ethics committee (Sweden). All subjects gave written informed consent in accordance with the Declaration of Helsinki. The protocol was approved by the above agencies.

## Author Contributions

Study conception and design: NA, J-MJ, MN, and IH. Collection and assembly of data: NA, J-MJ, KB, AE, SW-F, AM, and SM. Data analysis and interpretation: NA, J-MJ, KB, AE, SW-F, AM, and IH. Manuscript writing: NA, J-MJ, and IH. Final approval of manuscript: all authors.

## Conflict of Interest Statement

The authors declare that the research was conducted in the absence of any commercial or financial relationships that could be construed as a potential conflict of interest.

## Footnotes

^1^http://bioinformatics.psb.ugent.be/webtools/Venn/.

^2^http://www.ogt.co.uk/products/915_sureseq_solid_tumour_panel.

^3^https://github.com/chapmanb/bcbio-nextgen.
