# Supplementary figures and images for: Involvement of Chromatin Remodeling Genes and the Rho GTPases RhoB and CDC42 in Ovarian Clear Cell Carcinoma
Source: Front Oncol. 2017 May 29;7:109. doi: 10.3389/fonc.2017.00109 (PMC5447048; doi:10.3389/fonc.2017.00109)

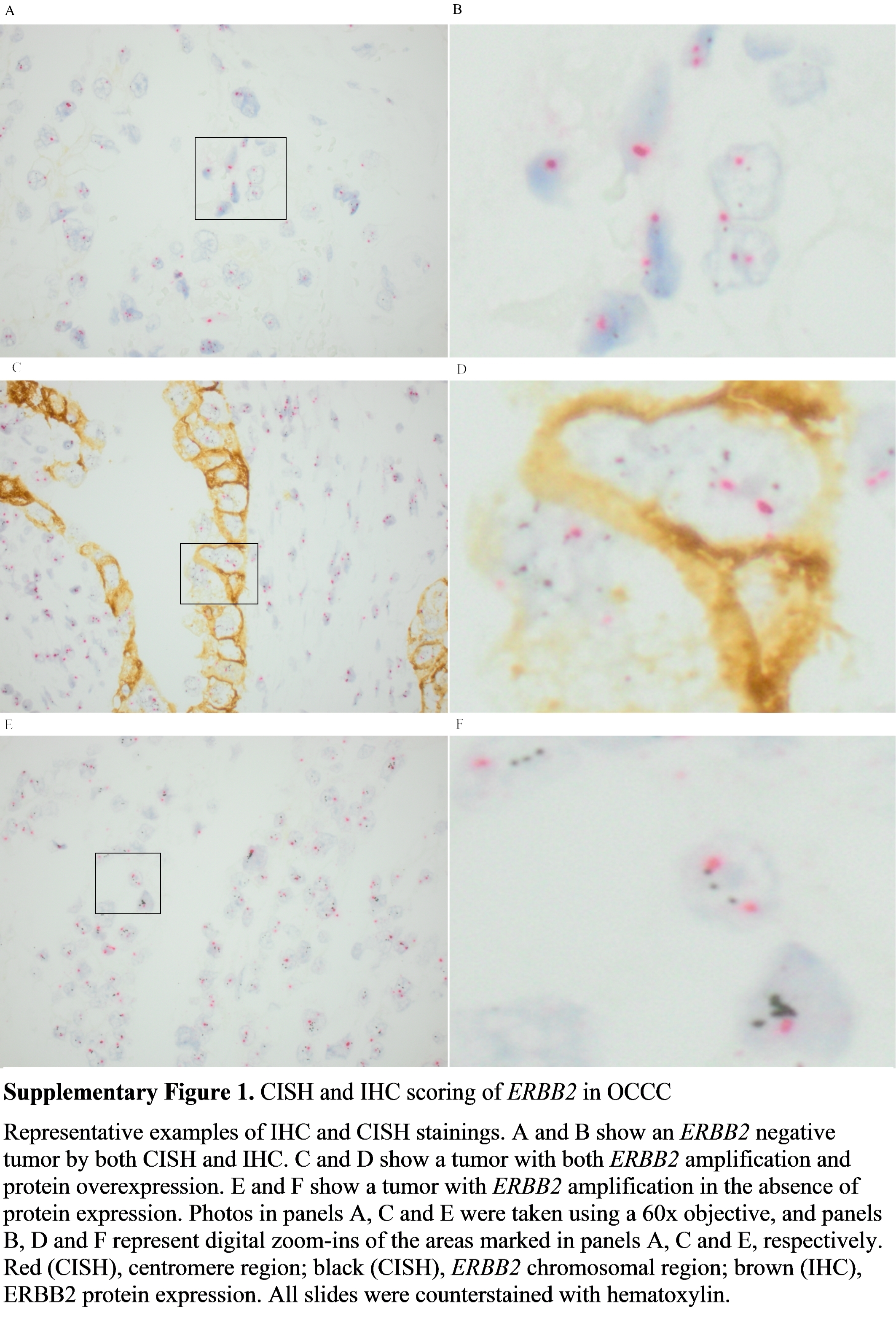

Supplement: Supplementary file 1 [file image_1.jpeg]
