# Supplementary material for: Involvement of Chromatin Remodeling Genes and the Rho GTPases RhoB and CDC42 in Ovarian Clear Cell Carcinoma
Source: Front Oncol. 2017 May 29;7:109. doi: 10.3389/fonc.2017.00109 (PMC5447048; doi:10.3389/fonc.2017.00109)

Supplementary File 2.

Boxplots of log2 significant ($P<0.05$) differentially expressed genes in selected pathways. Kruskal-Wallis test was used for significance test between subgroups. C: Clear Cell, E: Endometrioid, M: Mucinous, S: Serous.

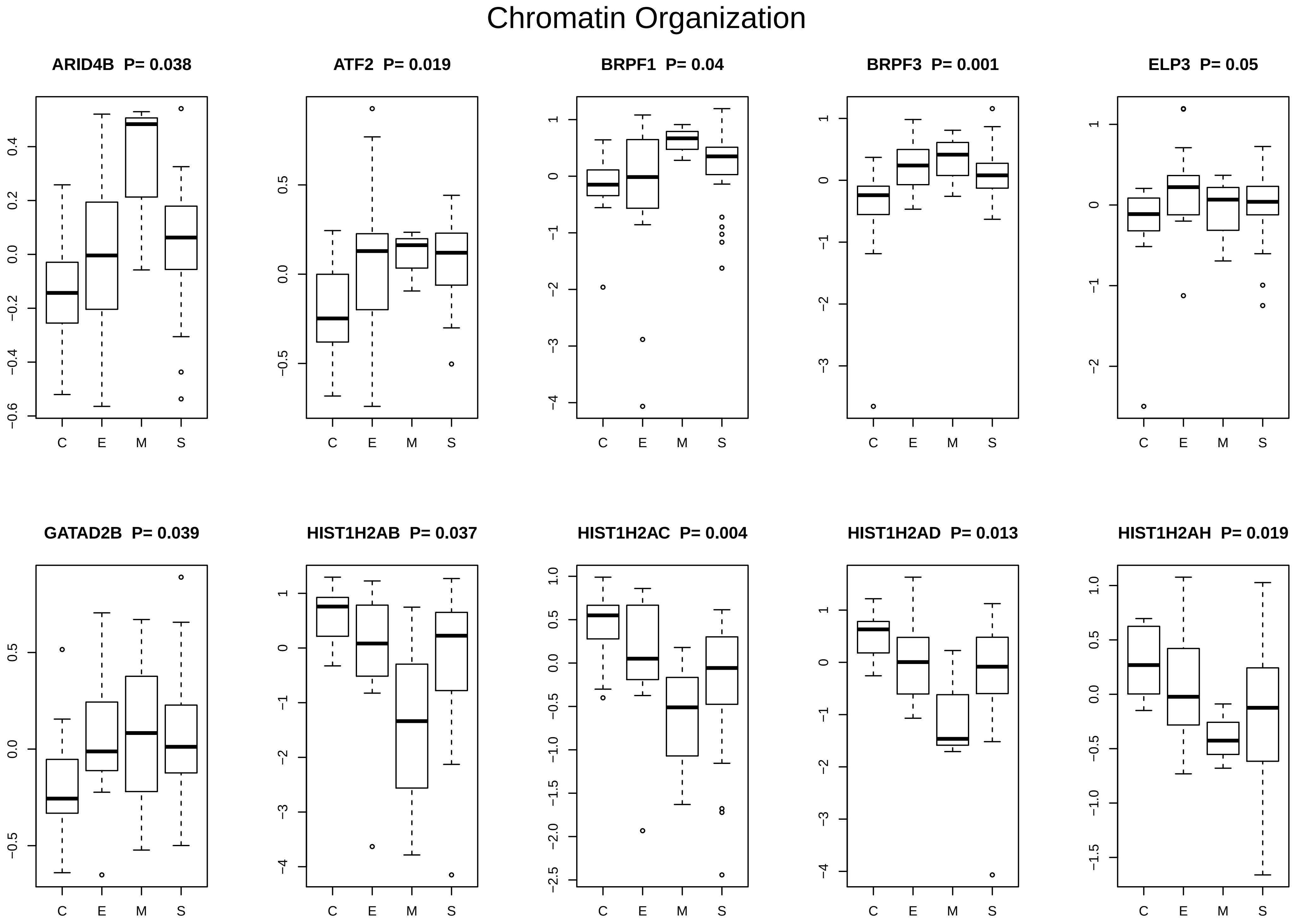
Chromatin Organization
ARID4B P= 0.038
ATF2 P= 0.019
BRPF1 P= 0.04
BRPF3 P= 0.001
ELP3 P= 0.05
GATAD2B P= 0.039
HIST1H2AB P= 0.037
HIST1H2AC P= 0.004
HIST1H2AD P= 0.013
HIST1H2AH P= 0.019
C E M S

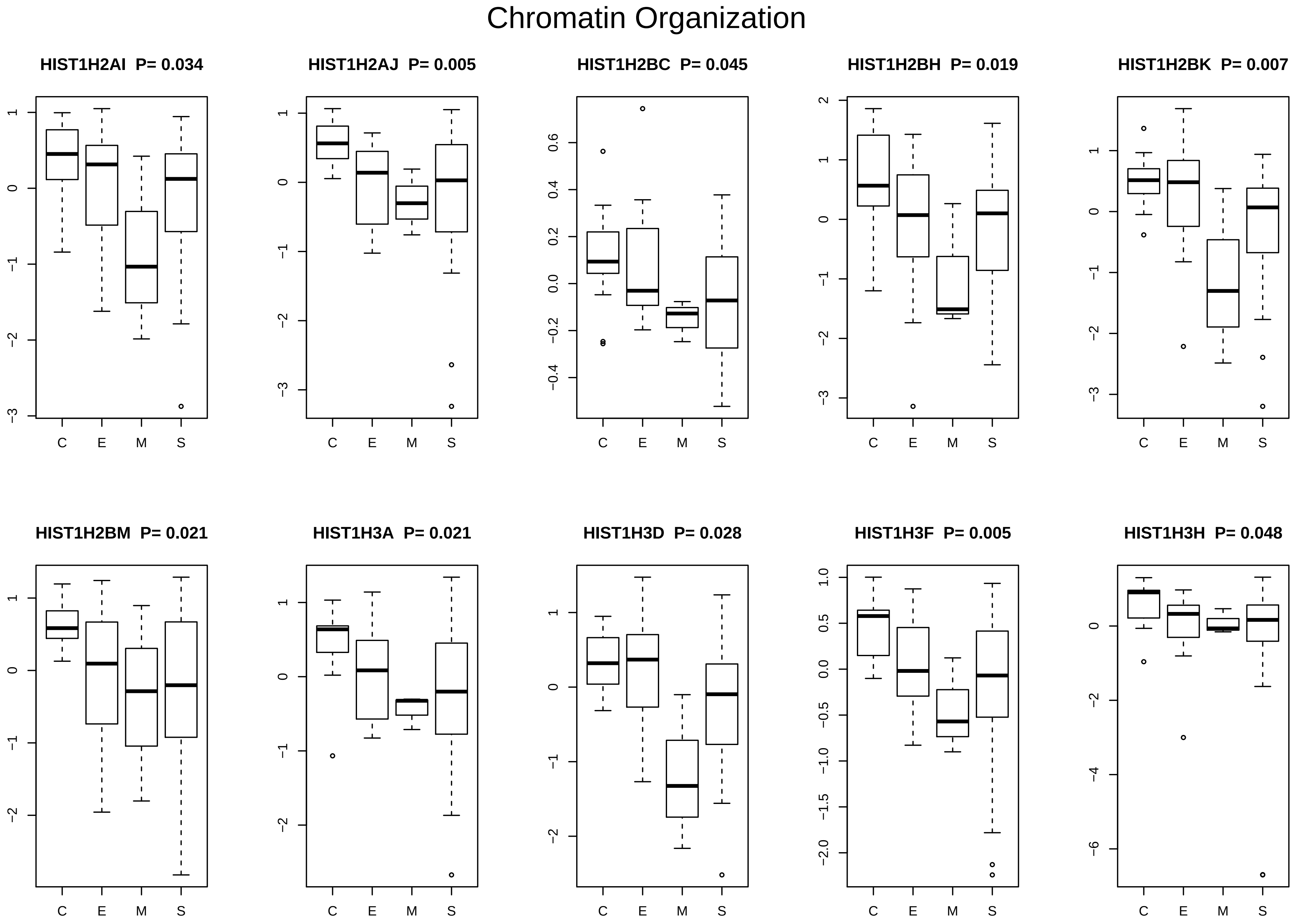
Chromatin Organization
HIST1H2AI P= 0.034
HIST1H2AJ P= 0.005
HIST1H2BC P= 0.045
HIST1H2BH P= 0.019
HIST1H2BK P= 0.007
HIST1H2BM P= 0.021
HIST1H3A P= 0.021
HIST1H3D P= 0.028
HIST1H3F P= 0.005
HIST1H3H P= 0.048
C
E
M
S

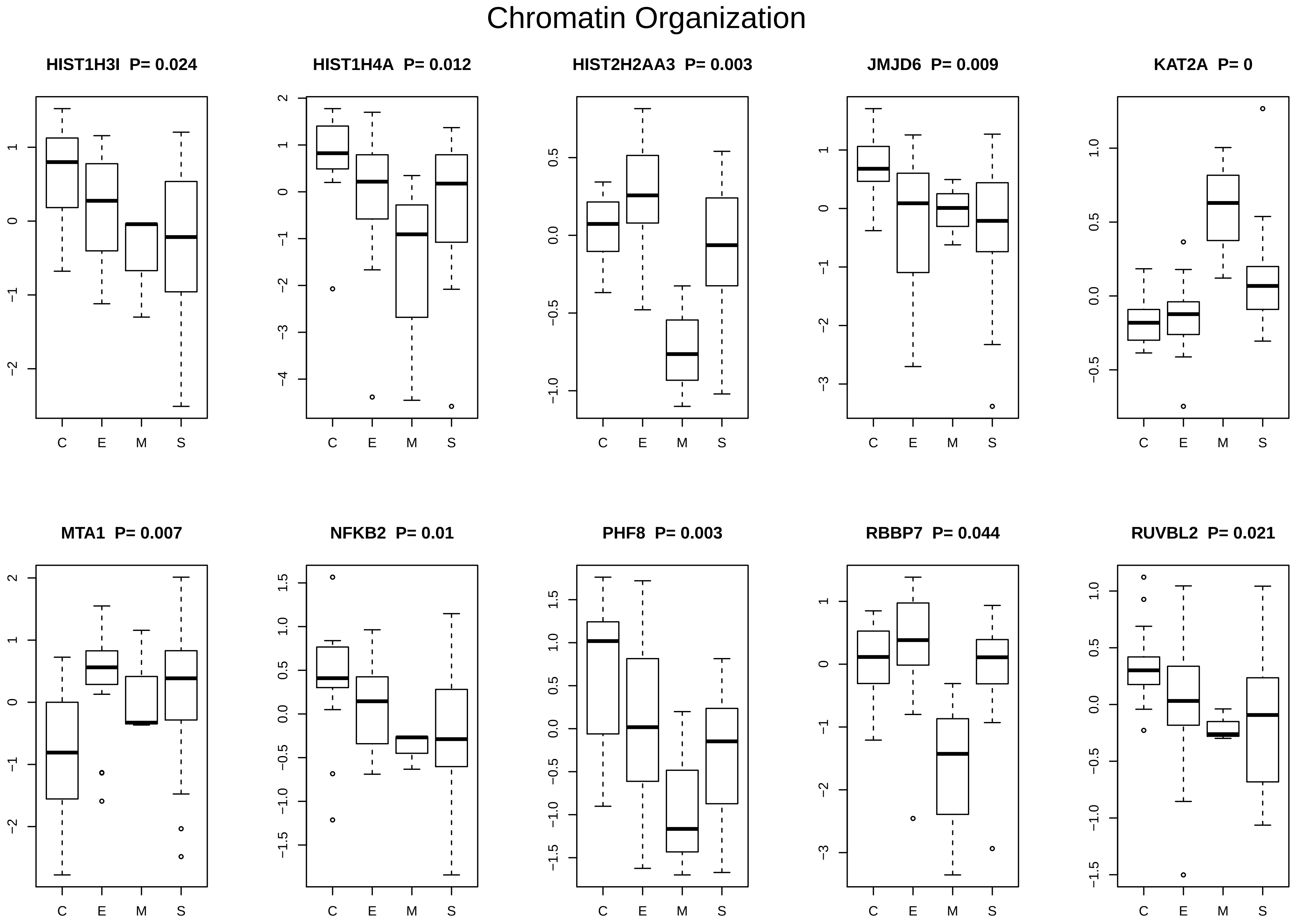
Chromatin Organization
HIST1H3I P= 0.024
HIST1H4A P= 0.012
HIST2H2AA3 P= 0.003
JMJD6 P= 0.009
KAT2A P= 0
MTA1 P= 0.007
NFKB2 P= 0.01
PHF8 P= 0.003
RBBP7 P= 0.044
RUVBL2 P= 0.021
C
E
M
S

Chromatin Organization

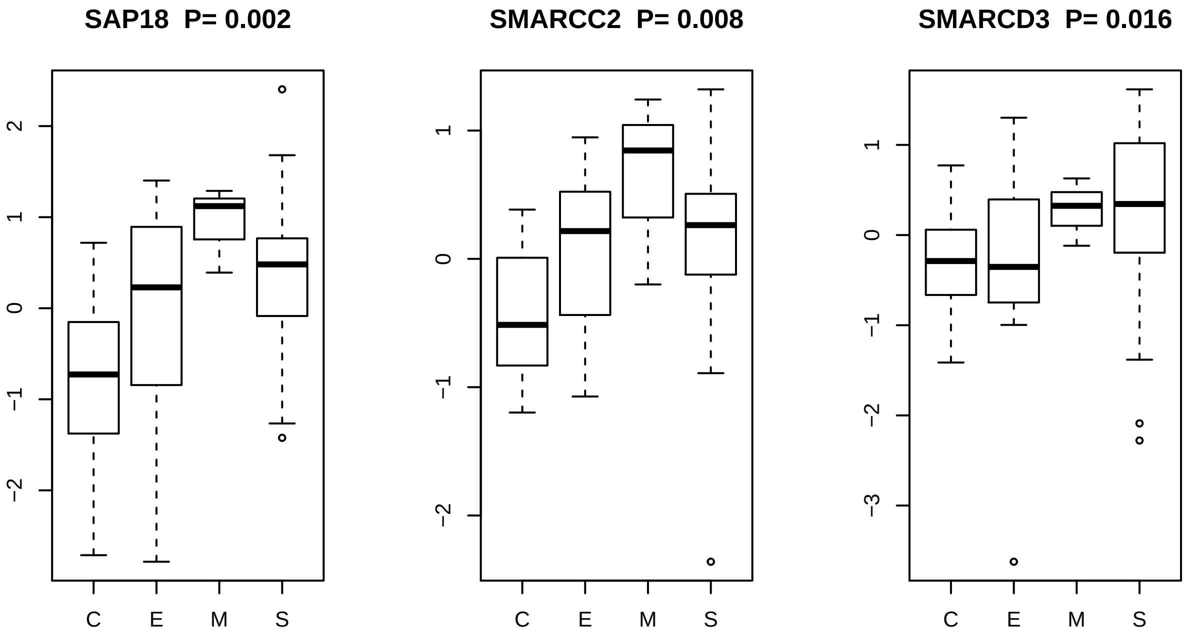

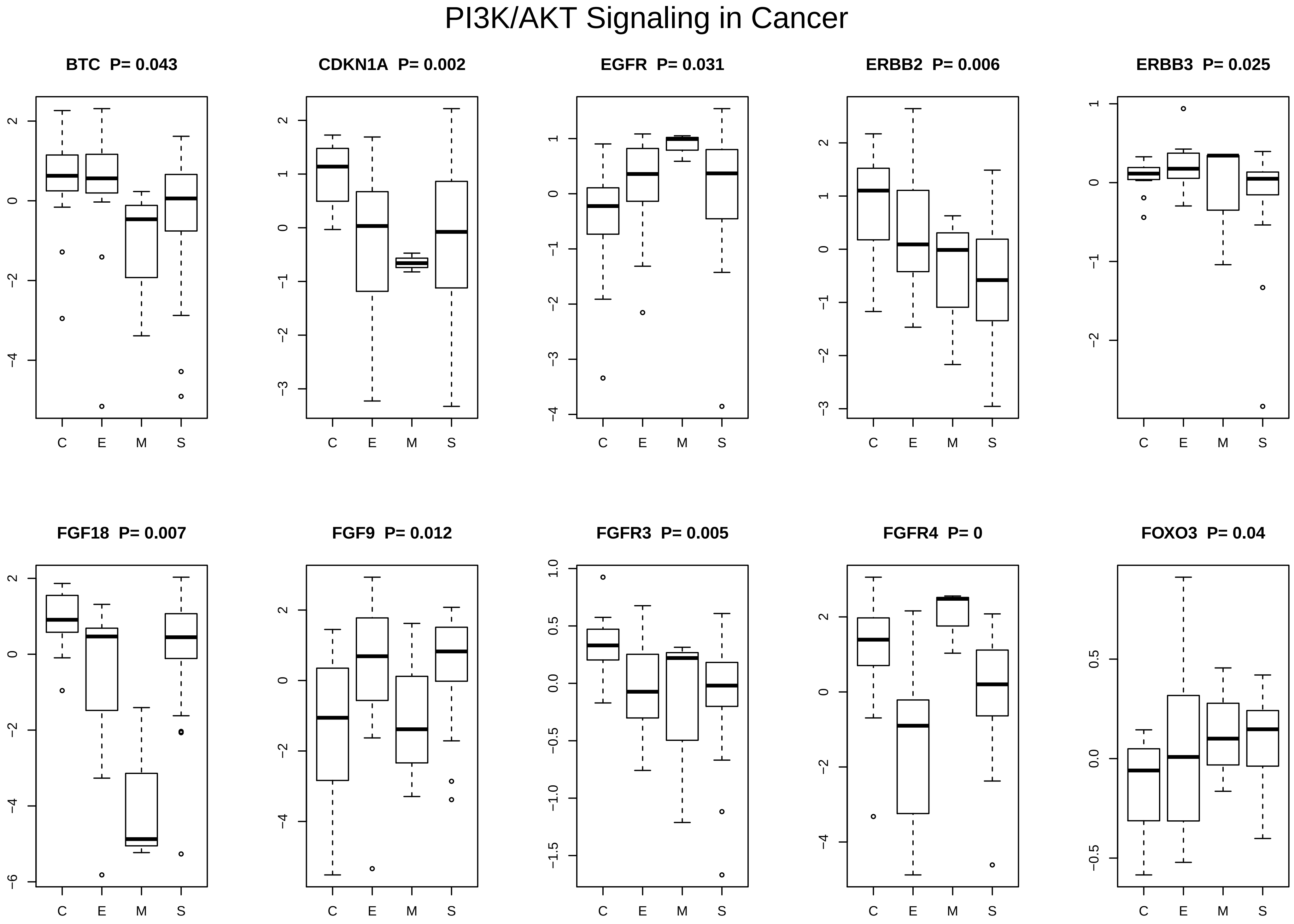
PI3K/AKT Signaling in Cancer
BTC P= 0.043
CDKN1A P= 0.002
EGFR P= 0.031
ERBB2 P= 0.006
ERBB3 P= 0.025
FGF18 P= 0.007
FGF9 P= 0.012
FGFR3 P= 0.005
FGFR4 P= 0
FOXO3 P= 0.04
C
E
M
S

PI3K/AKT Signaling in Cancer

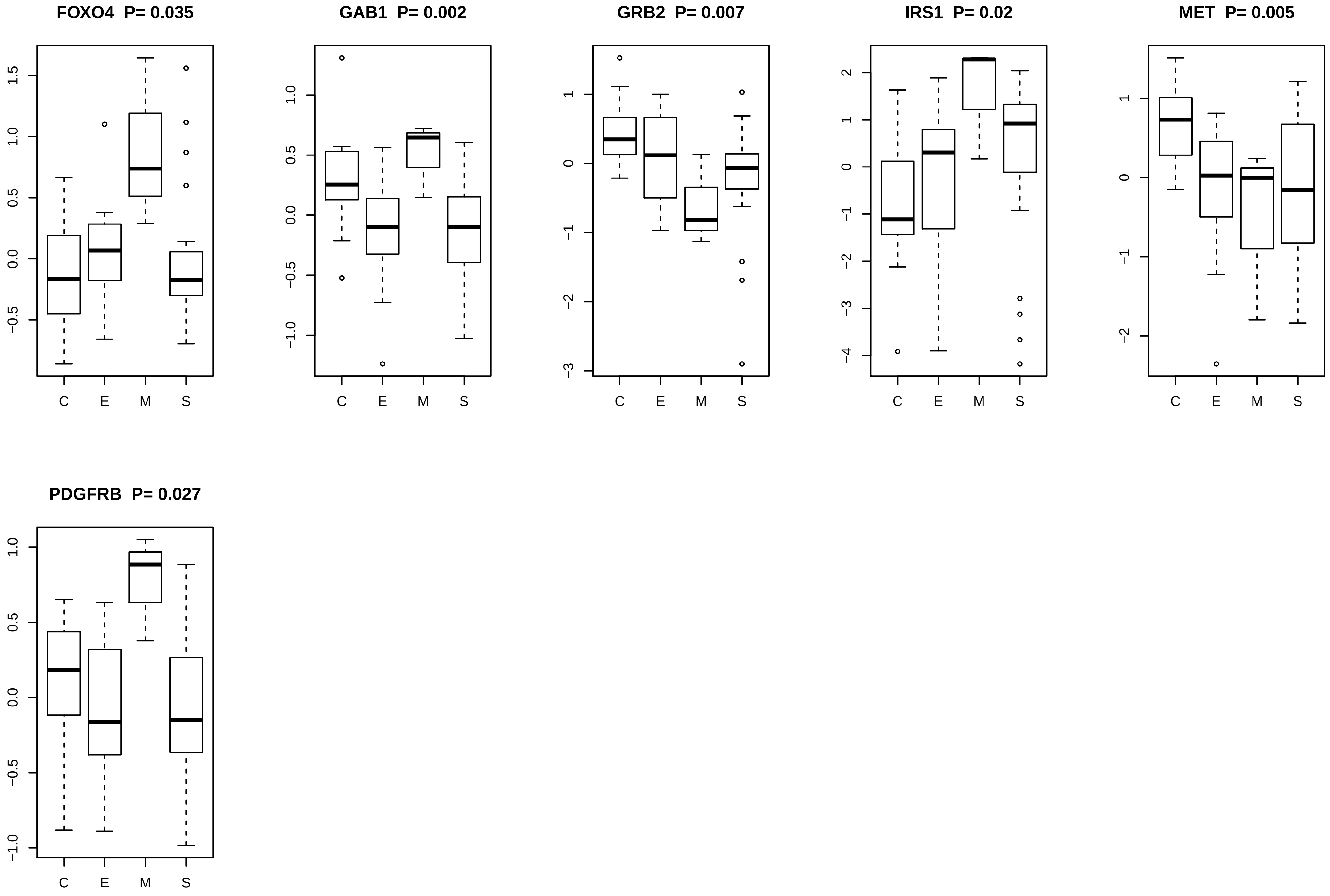

RAF MAP kinase cascade

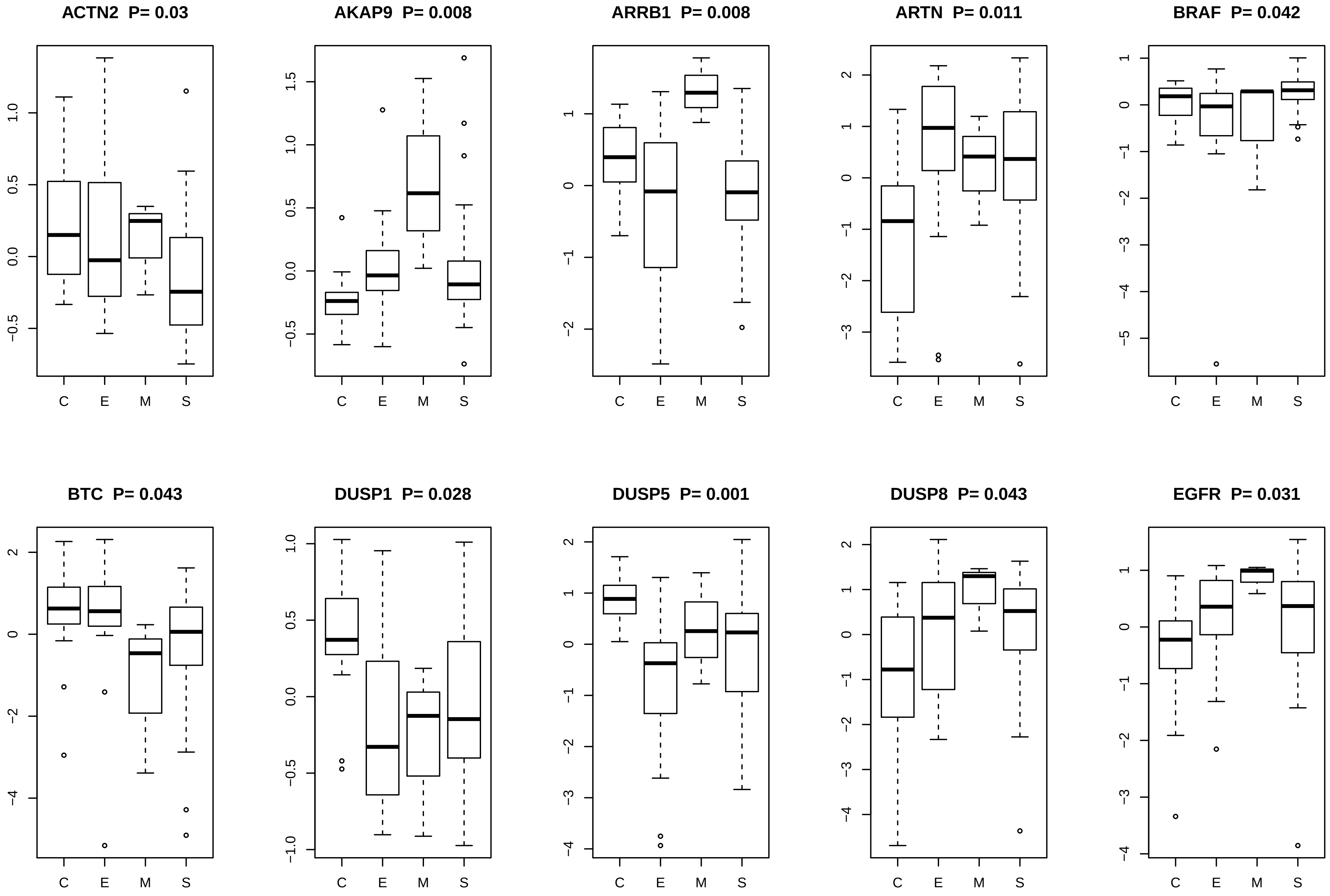

RAF MAP kinase cascade

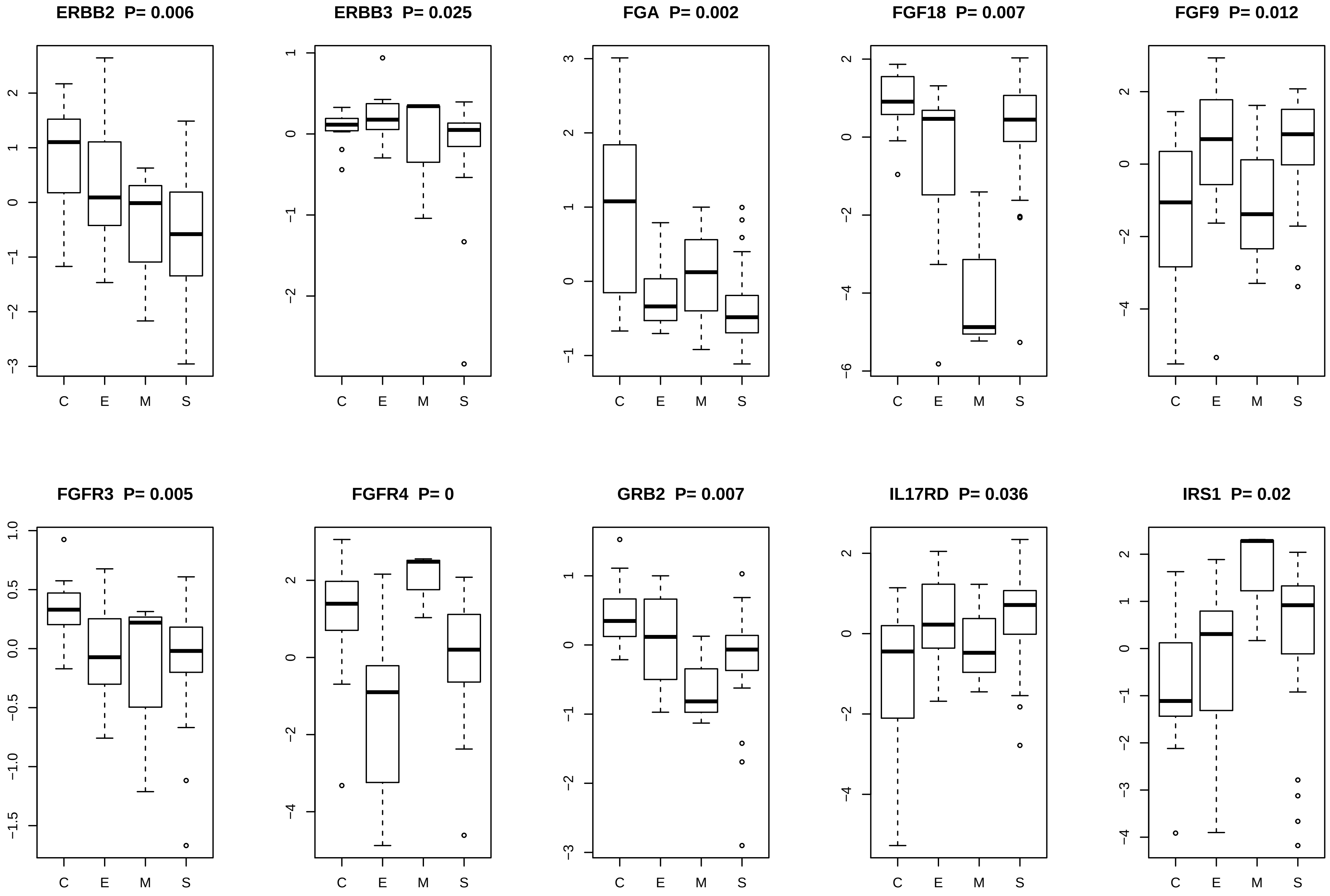

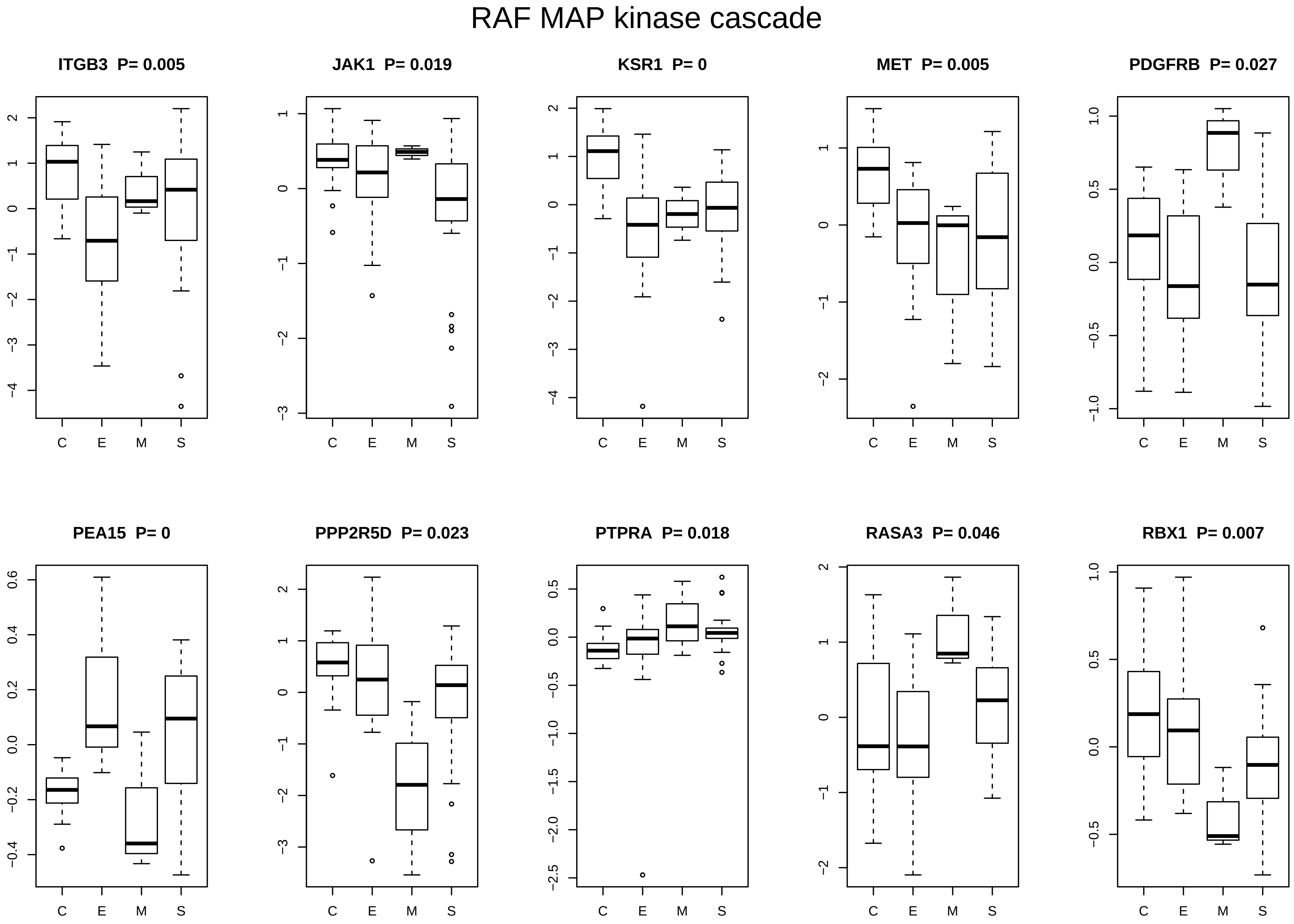
RAF MAP kinase cascade
ITGB3 P= 0.005
JAK1 P= 0.019
KSR1 P= 0
MET P= 0.005
PDGFRB P= 0.027
PEA15 P= 0
PPP2R5D P= 0.023
PTPRA P= 0.018
RASA3 P= 0.046
RBX1 P= 0.007
C
E
M
S

RAF MAP kinase cascade

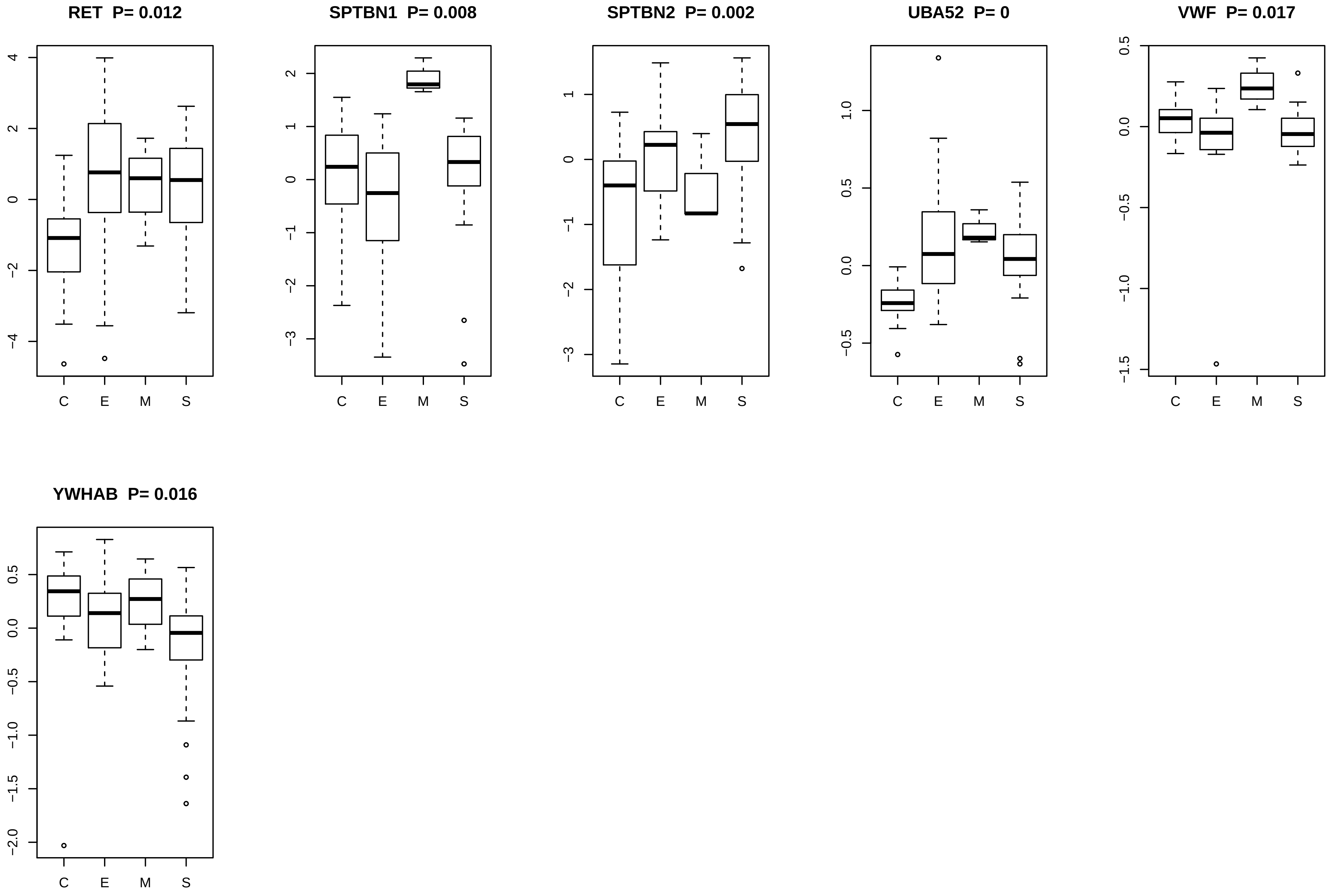

Rho GTPase Cycle

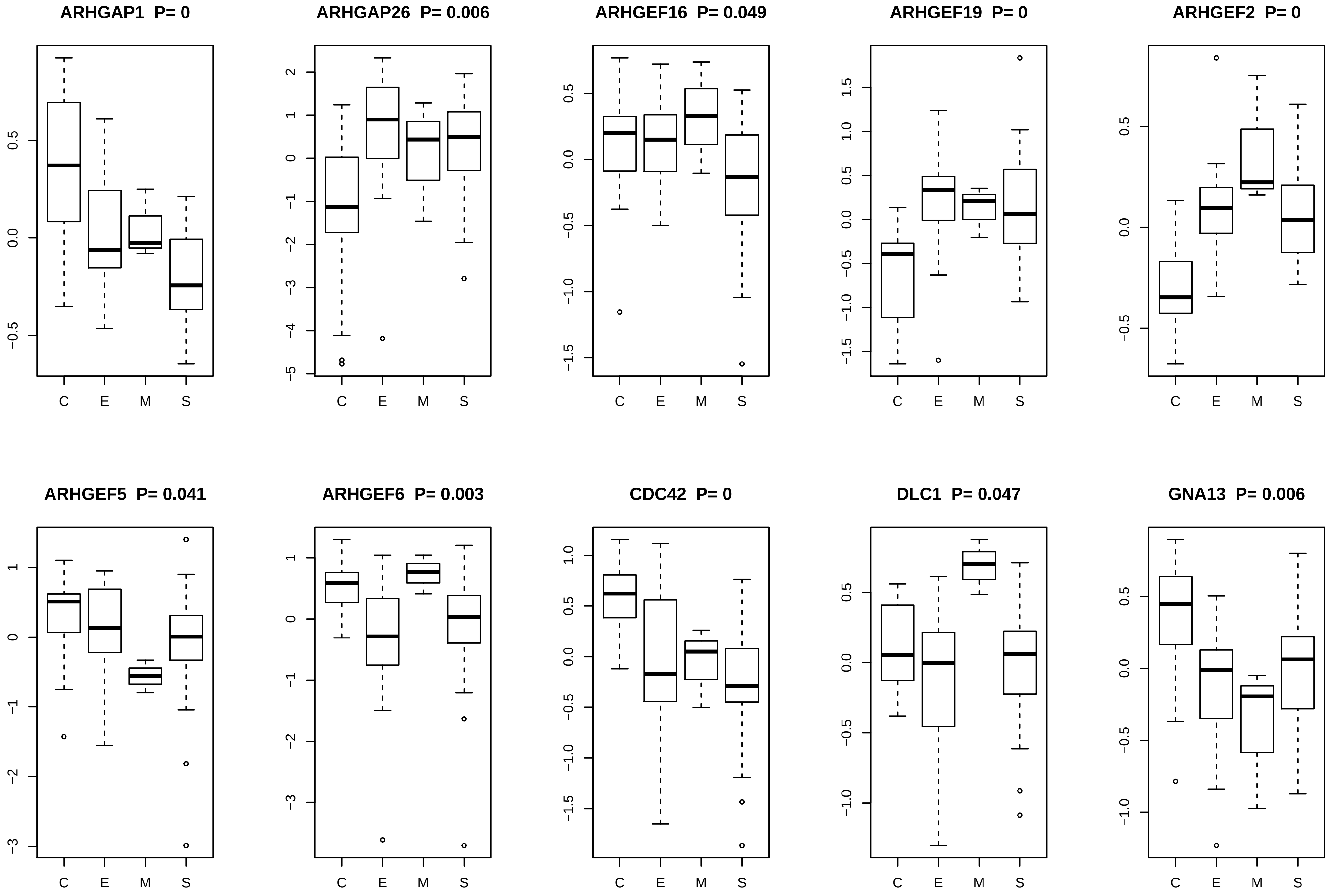

Rho GTPase Cycle

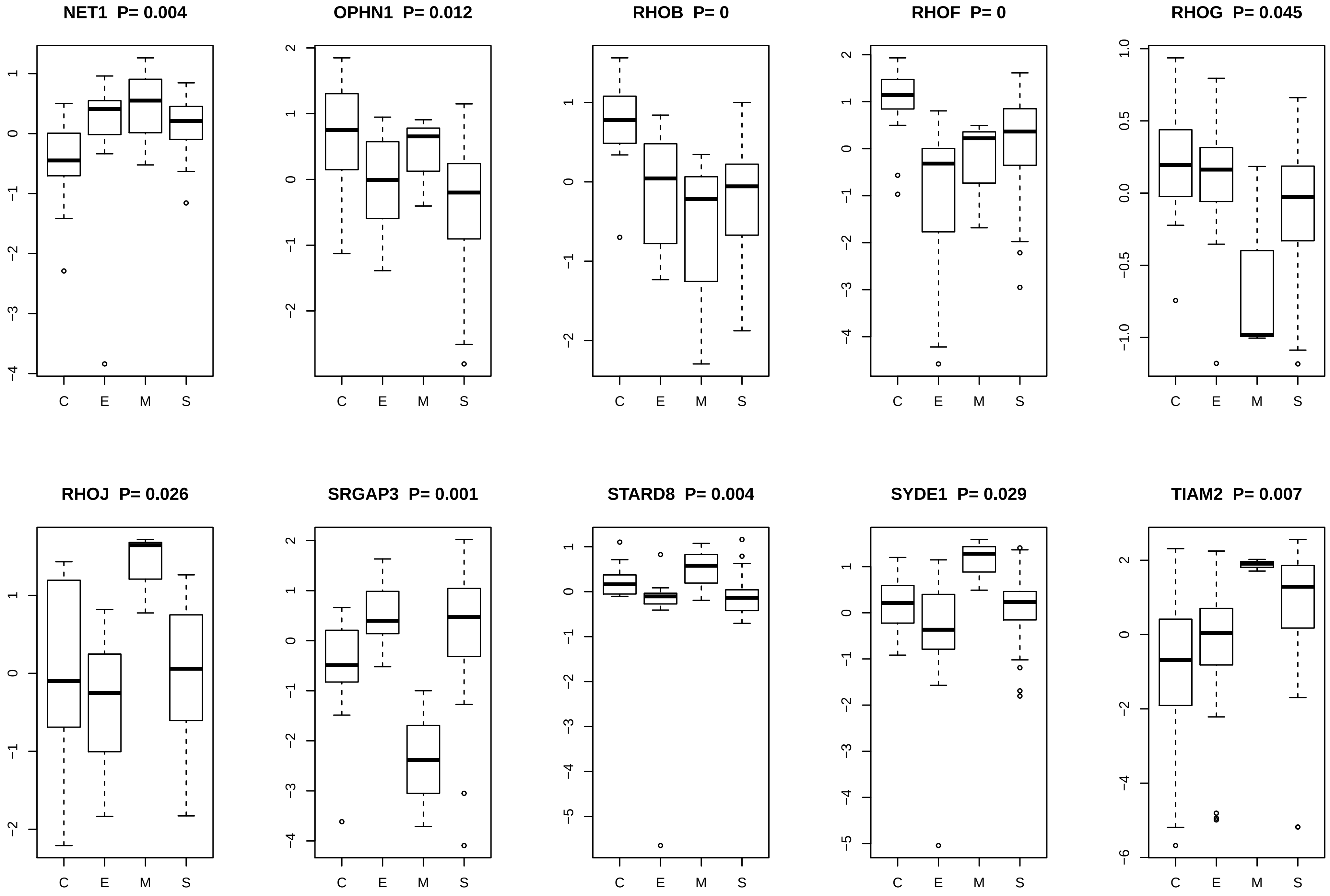

Rho GTPase Cycle

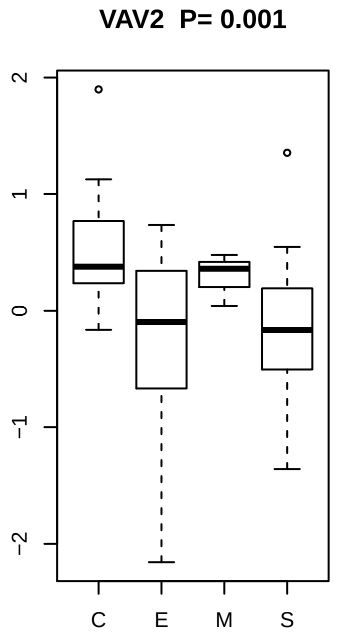

Transcriptional regulation by the AP2/TFAP2 family of transcription factors

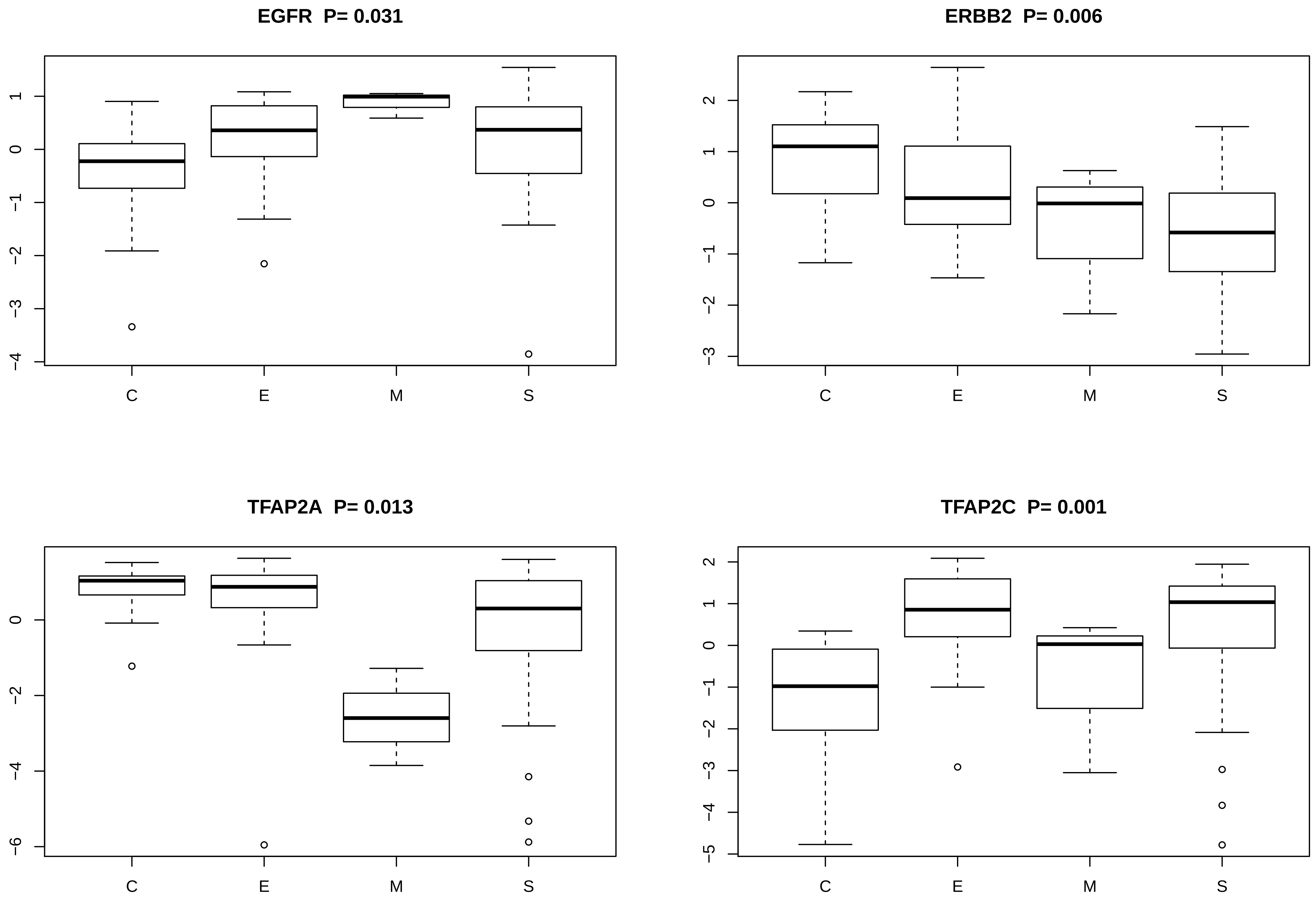

Supplement: Supplementary file 3 [file data_sheet_2.pdf]
