# Supplementary material for: Involvement of Chromatin Remodeling Genes and the Rho GTPases RhoB and CDC42 in Ovarian Clear Cell Carcinoma
Source: Front Oncol. 2017 May 29;7:109. doi: 10.3389/fonc.2017.00109 (PMC5447048; doi:10.3389/fonc.2017.00109)
Supplement: Supplementary file 4 [file data_sheet_3.docx]

**Supplementary Table S1.** Genes included in the Oxford Gene Technology™ Solid Tumor Panel

| *AKT1* | *BRCA2* | *FGFR2* | *MAP2K1* | *NOTCH1* | *SMAD4* |
| --- | --- | --- | --- | --- | --- |
| *ALK* | *CDH1* | *FGFR3* | *MED12* | *NRAS* | *SMARCA4* |
| *APC* | *CDKN1B* | *FOXA1* | *MET* | *PDGFRA* | *SMARCB1* |
| *AR* | *CDKN2A* | *GNAS* | *MLH1* | *PDGFRB* | *SMO* |
| *ARID1A* | *CHD1* | *HRAS* | *KMT2A (MLL1)* | *PIK3CA* | *SPOP* |
| *ASXL1* | *CTNNB1* | *IDH1* | *KMT2C (MLL3)* | *PTEN* | *STK11* |
| *ATM* | *DDR2* | *JAK2* | *KMT2D (MLL2)* | *PTPN11* | *TP53* |
| *AXL* | *EGFR* | *JAK3* | *MTOR* | *RB1* | *KDM6A (UTX)* |
| *BRAF* | *ERBB2* | *KIT* | *NF1* | *RET* | *VHL* |
| *BRCA1* | *FGFR1* | *KRAS* | *NKX3.1* | *ROS1* | *ZFHX3* |

**Supplementary Table S2.** GO terms with a semantic comparison score >0.683 between the present study and the study by Zorn *et al.* using the G-SESAME online tool

| **GO term ID** | **Description** |
| --- | --- |
| GO:0048856 | anatomical structure development |
| GO:0048646 | anatomical structure formation involved in morphogenesis |
| GO:0009653 | anatomical structure morphogenesis |
| GO:0065007 | biological regulation |
| GO:0008150 | biological_process |
| GO:0001568 | blood vessel development |
| GO:0072358 | cardiovascular system development |
| GO:0007154 | cell communication |
| GO:0030154 | cell differentiation |
| GO:0007166 | cell surface receptor signaling pathway |
| GO:0048869 | cellular developmental process |
| GO:0009987 | cellular process |
| GO:0070887 | cellular response to chemical stimulus |
| GO:0071495 | cellular response to endogenous stimulus |
| GO:0032870 | cellular response to hormone stimulus |
| GO:0071310 | cellular response to organic substance |
| GO:0051716 | cellular response to stimulus |
| GO:0072359 | circulatory system development |
| GO:0032502 | developmental process |
| GO:0060429 | epithelium development |
| GO:0007275 | multicellular organismal development |
| GO:0048519 | negative regulation of biological process |
| GO:0008285 | negative regulation of cell proliferation |
| GO:0048523 | negative regulation of cellular process |
| GO:0009892 | negative regulation of metabolic process |
| GO:0007399 | nervous system development |
| GO:0048513 | organ development |
| GO:0048518 | positive regulation of biological process |
| GO:0045597 | positive regulation of cell differentiation |
| GO:0048522 | positive regulation of cellular process |
| GO:0051094 | positive regulation of developmental process |
| GO:0009893 | positive regulation of metabolic process |
| GO:0050789 | regulation of biological process |
| GO:0045595 | regulation of cell differentiation |
| GO:0042127 | regulation of cell proliferation |
| GO:0050794 | regulation of cellular process |
| GO:0050793 | regulation of developmental process |
| GO:0032879 | regulation of localization |
| GO:0019222 | regulation of metabolic process |
| GO:0065009 | regulation of molecular function |
| GO:0051239 | regulation of multicellular organismal process |
| GO:0051147 | regulation of muscle cell differentiation |
| GO:0048583 | regulation of response to stimulus |
| GO:0009719 | response to endogenous stimulus |
| GO:0050896 | response to stimulus |
| GO:0007165 | signal transduction |
| GO:0023052 | signaling |
| GO:0044700 | single organism signaling |
| GO:0044707 | single-multicellular organism process |
| GO:0044763 | single-organism cellular process |
| GO:0044767 | single-organism developmental process |
| GO:0044699 | single-organism process |
| GO:0048731 | system development |
| GO:0009888 | tissue development |
| GO:0035295 | tube development |
| GO:0001944 | vasculature development |
